# Four new species of *Hexanchorus* Sharp from Ecuador (Coleoptera, Elmidae) with DNA barcoding and notes on the distribution of the genus

**DOI:** 10.3897/zookeys.838.33086

**Published:** 2019-04-11

**Authors:** Marek Linský, Zuzana Čiamporová-Zaťovičová, Fedor Čiampor Jr

**Affiliations:** 1 Department of Zoology, Faculty of Natural Science, Comenius University, Mlynská dolina B-1, SK-84215 Bratislava, Slovakia Comenius University Bratislava Slovakia; 2 Zoology Lab, Plant Science and Biodiversity Centre, Slovak Academy of Sciences, Dúbravská cesta 9, SK-84523, Bratislava, Slovakia Plant Science and Biodiversity Centre, Slovak Academy of Sciences Bratislava Slovakia

**Keywords:** Andes, diversity, Larainae, Latin America, mtDNA, riffle beetles, new record

## Abstract

The riffle beetle genus *Hexanchorus* Sharp, 1882 is distributed from Mexico to Argentina, forming an important component of the freshwater invertebrate fauna of Latin America. With 21 described species, *Hexanchorus* represents one of the most speciose Larainae genera, but its real diversity is likely much higher. We analysed material from a relatively small area in Ecuador, resulting in the first record of *H.cordillierae* for Ecuador and discovery of four new species and one subspecies: *Hexanchorusvirilis***sp. n.**, *Hexanchorusrostratus***sp. n.**, *Hexanchorusshepardi***sp. n.**, *Hexanchorusonorei***sp. n.** and *Hexanchorusonoreisagittatus***ssp. n.** For delimiting and characterizing species, both morphological and molecular (mtCOI DNA barcodes) data were used. A distribution map of *Hexanchorus* species is provided based on published records.

## Introduction

The Neotropics represent one of the most life-rich regions in the world. With its enormously diverse ecosystems from large lowlands, through Amazonian rainforests up to the snow-covered peaks of the Andes, it provides manifold living conditions suitable for an inordinate number of various organisms. However, many taxonomic groups inhabiting the Neotropics are still very poorly known including the riffle beetles, despite numerous recently published taxonomic papers describing their diversity (e.g. Maier 2013, Čiampor Jr et al. 2017, Martinéz Román et al. 2017, Polizei and Barclay 2018).

Ecuador is a relatively small country, but due to its great altitudinal variation and the presence of rainforests, it belongs to the top ten most biodiverse countries. The Elmidae of this region were studied mostly by Delève in the 1960s, who recorded 23 species in nine genera (Delève 1968). After forty-five years, these numbers increased to 59 species in 19 genera (Monte and Mascagni 2012).

The genus *Hexanchorus* Sharp, 1882, with 21 known species, is the largest and most likely the most wide-spread genus of Larainae in the Neotropics. The area of its distribution reaches from Mexico through Central America and the West Indies up to northern Argentina (Jäch et al. 2016). In contrast to its great distribution, 1/3 of all known species (seven) can be found in one country. This is almost certainly biased by uneven distribution of the research, pointing to our insufficient knowledge of the *Hexanchorus* fauna from the other countries and probably also to the large diversity of the genus. Here we processed the *Hexanchorus* material from Ecuador, collected at several of the 50 sites surveyed in 2013, including fresh material used for DNA barcode analyses to characterise species.

## Material and methods

The studied material was collected by net sampling in small streams flowing in primary or degraded forest or at light. Specimens were fixed in pure alcohol directly in the field. The majority of material was collected in Ecuador. Additional specimens come from two localities in Venezuela and one site in Brazil. For the morphological study, specimens were cleaned and examined under a Leica M205C stereomicroscope at magnifications up to 160×. Male genitalia were studied as temporary glycerine slides at magnifications up to 600×, using a Leica DM1000 light microscope. Drawings were made with a drawing tube, subsequently scanned and finalized in Adobe Photoshop CS5. Habitus photographs were made using a Leica M205C with a Nikon D3s digital camera attached. Morphological terms generally follow Kodada et al. (2016).

Morphometric characters were measured with an ocular grid to the nearest 0.05 mm. Abbreviations used in the text: **CL** – body length (measured from the anterior margin of the pronotum to the elytral apices), **EL** – elytral length, **EW** – maximum elytral width, **PL** – pronotal length, **PW** – maximum pronotal width, **NMW** – Natural History Museum (Vienna, Austria), **CCB** – collection of Fedor Čiampor Jr (Bratislava, Slovakia), **PUCE** – collection of the Pontifical Catholic University of Ecuador (Quito, Ecuador), **MNHN** – National Museum of Natural History (Paris, France), **RBINS** – Royal Belgian Institute of Natural Sciences (Brussels, Belgium). All type specimens belong to PUCE, but are presently on long-term loan at the CCB.

For the DNA analyses, 26 adults of *Hexanchorus* and 3 adults of related Larainae species were used. The dataset is available on https://doi.org/10.5883/DS-ELMHEXAN. DNA was isolated from the whole specimens using DNeasy Blood and Tissue Kit (Qiagen) according to manufacturer’s protocol or by phenol-chlorophorm extraction method. A fragment of the 5’ end of the mitochondrial gene for cytochrome c oxidase subunit I (COI) was amplified with primers LCO1490, HCO2198 (Folmer et al. 1994). Amplification products were purified by alkaline phosphatase (FastAP) and exonuclease and sequenced from both sides in Macrogen Europe Inc. (Amsterdam, Netherlands). Raw sequences were assembled and edited in Sequencher v5.1. The genetic distances were measured using K2P model, maximum likelihood tree and bootstrap support was performed in MEGA software v7 (Kumar et al. 2016). The best-fitted substitution model (GTR+I+G) was selected by jModelTest 2 (Darriba et al. 2012). Species delimitation (bPTP, mPTP, ABGD) was run on servers (http://species.h-its.org, http://mptp.h-its.org/#/tree, https://wwwabi.snv.jussieu.fr/public/abgd/abgdweb.html) with default settings. For outgroup rooting, sequences of *Potamophilopsbostrychophallus* Maier, 2013, *Pseudodisersusgoudotii* (Guérin-Méneville, 1843) and *Disersusinca* Spangler & Santiago, 1987 were used. The final tree was edited in FigTree v1.4.2 and Adobe Illustrator CS5. Vouchers are deposited in the CCB, and sequences were sent to GenBank and BOLD (accession numbers and BINs are in Table 1).

**Table 1.** Samples used in the molecular analyses: location of samples, GenBank and BOLD Data Systems BIN accession numbers. (FZ numbers refer to the vouchers used for DNA extraction)

**Sample**	**Location**	**GenBank no.**	**BOLD BIN no.**
*Hexanchoruscordillierae* FZ0602	Ecuador, Napo	MK155275	BOLD:ADO9755
*Hexanchoruscordillierae* FZ0956	Ecuador, Napo	MK155252	BOLD:ADO9755
*Hexanchoruscordillierae* FZ0966	Ecuador, Pastaza	MK155257	BOLD:ADO9755
*Hexanchoruscordillierae* FZ0972	Ecuador, Napo	MK155265	BOLD:ADO9755
*Hexanchoruscordillierae* FZ0987	Ecuador, Pastaza	MK155279	BOLD:ADO9755
*Hexanchoruscordillierae* FZ1242	Ecuador, Pastaza	MK155280	BOLD:ADO9755
*Hexanchoruscordillierae* FZ1243	Ecuador, Pastaza	MK155271	BOLD:ADO9755
*Hexanchoruscordillierae* FZ1244	Ecuador, Napo	MK155282	BOLD:ADO9755
*Hexanchoruscordillierae* FZ1245	Ecuador, Napo	MK155270	BOLD:ADO9755
*Hexanchoruscordillierae* FZ1248	Ecuador, Napo	MK155277	BOLD:ADO9755
*Hexanchorusonoreisagittatus* FZ0773	Ecuador, Morona-Santiago	MK155259	BOLD:ADB7879
*Hexanchorusonoreisagittatus* FZ0970	Ecuador, Morona-Santiago	MK155262	BOLD:ADB7879
*Hexanchorusonoreisagittatus* FZ1252	Ecuador, Morona-Santiago	MK155261	BOLD:ADB7879
*Hexanchorusonoreionorei* FZ0621	Ecuador, Morona-Santiago	MK155268	BOLD:ADB7879
*Hexanchorusonoreionorei* FZ0969	Ecuador, Morona-Santiago	MK155263	BOLD:ADB7879
*Hexanchorusonoreionorei* FZ0971	Ecuador, Morona-Santiago	MK155258	BOLD:ADB7879
*Hexanchorusonoreionorei* FZ1246	Ecuador, Morona-Santiago	MK155256	BOLD:ADB7879
*Hexanchorusshepardi* FZ0967	Ecuador, Napo	MK155264	BOLD:ADO9756
*Hexanchorusshepardi* FZ0968	Ecuador, Napo	MK155276	BOLD:ADO9756
*Hexanchorusshepardi* FZ1247	Ecuador, Napo	MK155267	BOLD:ADO9756
*Hexanchorusvirilis* FZ0623	Ecuador, Pastaza	MK155281	BOLD:ADB7877
*Hexanchorusvirilis* FZ0960	Ecuador, Pastaza	MK155251	BOLD:ADB7877
*Hexanchorusvirilis* FZ1241	Ecuador, Pastaza	MK155253	BOLD:ADB7877
*Hexanchorusvirilis* FZ1250	Ecuador, Pastaza	MK155266	BOLD:ADB7877
*Hexanchorustarsalis* FZ1249	Brazil, Rio Grande do Sul	MK155255	BOLD:ADO9167
*Hexanchorustarsalis* FZ1253	Brazil, Rio Grande do Sul	MK155278	BOLD:ADO9167
*Potamophilopsbostrychophallus* FZ0383	Venezuela, El Caura	MK155274	BOLD:ADB8887
*Pseudodisersusgoudotii* FZ0855	Ecuador, Pastaza	MK155254	BOLD:ADB9256
*Disersusinca* FZ0788	Ecuador, Morona-Santiago	MK155273	BOLD:ADB8448

## Results

### Molecular data analysis

Sequences of the barcoding fragment from 26 specimens were used in the analysis, representing six putative *Hexanchorus* species. Four of the five new species described herein are also included, amplification of COI failed for *H.rostratus* sp. n., likely due to degraded DNA. The final fragment was 625bp long with no ambiguous sites or indels. The maximum likelihood (ML) analysis revealed five distinct clades, separated by the genetic distance of 1.1–12.5% (Suppl. material 1: Table S1). Among *H.cordillierae*, *H.onorei* sp. n. and *H.shepardi* sp. n., smaller genetic distances were recorded, ranging from 1.1 to 2.3%. However, all clusters representing these species have robust support and the taxa proposed are further supported by delimitation analyses and distinct morphological characters. The lower genetic distance could be thus attributed to recent speciation. In *H.onorei* sp. n., two clearly distinguishable morphological forms were recovered, and easily recognized by the size and structure of the male genitalia. These forms described here as subspecies are represented by well-separated clades (molecular data) with robust support in the ML tree, but with very small genetic distance (0.3%).

## Taxonomy

### Hexanchorus cordillierae

Taxon classificationAnimaliaColeopteraElmidae

(Guérin Méneville, 1843)

**Figures 1–5.**
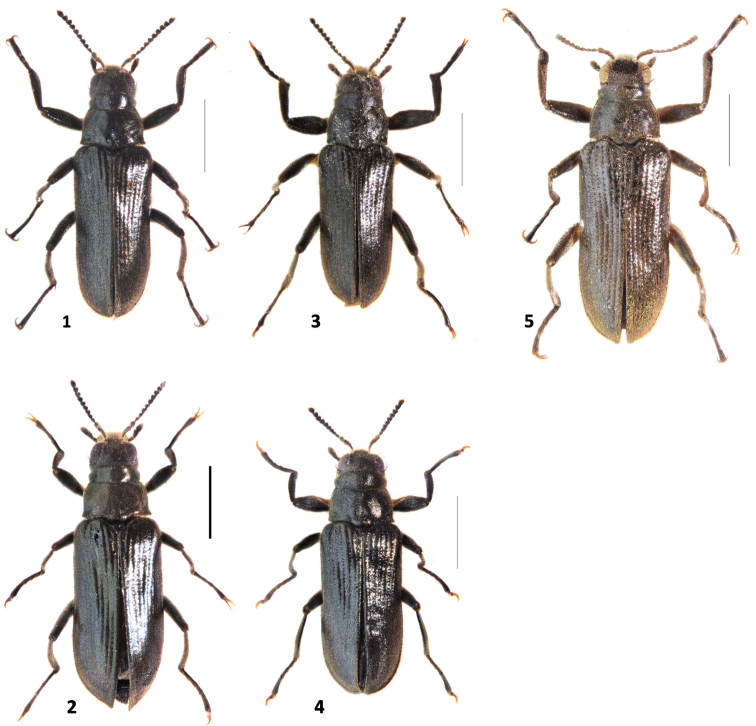
*Hexanchorus*habitus: **1***H.cordillierae* male **2***H.cordillierae* female **3***H.virilis* sp. n. male **4**H.cfvirilis sp. n. female **5***H.rostratus* sp. n. male. Scale: 1 mm.

Figs 1 , 2 , 12 , 13 , 23 , 24 , 36

#### Material examined.

(PUCE, NMW, CCB): 21 ♂♂, 10 ♀♀: “Ecuador, Napo prov., river near Don Napo ranch, Río Anzu, 01°14’17.2”S, 77°52’56.0”W 542m a.s.l., 13.8.2013, at light, Čiampor & Čiamporová-Zaťovičová lgt.”; 1 ♂ “Ecuador, Pastaza prov., 10 de Agosto env., 01°21‘37.1“S, 77°51‘55.7“W 900m a.s.l., 16.8.2013, stream ca 1m wide, above confluence with larger stream, fast flowing with boulders, Čiampor & Čiamporová-Zaťovičová lgt.”; 5 ♂♂, 5 ♀♀ “Ecuador, Napo prov., road to Coca, Sumaco env., 00°43‘29.0“S, 77°46‘01.4“W 1109m a.s.l., 17.8.2013, confluence of two larger streams, with gravel, stones, boulders, Čiampor & Čiamporová-Zaťovičová lgt.”; 5 ♂♂, 5 ♀♀ “Ecuador, Pastaza prov., Río Uklan, 01°17’13.8”S, 77°38’52.5”W 468m a.s.l., 18.8.2013, bigger river with lowland character, stream ca 15m wide, slow flowing with small riffles, with boulders, rock tables and sand, Čiampor & Čiamporová-Zaťovičová lgt.”.

#### Diagnosis.

*Hexanchoruscordillierae* can be distinguished from all species of the genus by a combination of the following male characters: 1) smaller size (CL: 2.96 – 3.16 mm); 2) mesotibiae with medial pubescent area long, reaching short of apex and lateral pubescent area long, extending to 2/3 of tibia; 3) mesotibiae with short thorn-like carina on inner apex; 4) metatibiae with feeble thorn-like carina on inner apex; 5) elytra with rounded apices; 6) fifth ventrite deeply and broadly emarginate; 7) aedeagus with right margin slightly dilated in middle in ventral view.

#### Redescription.

**Male.** Body elongate, subparallel, dorsum moderately convex (Fig. 1). Length (CL) 2.96 – 3.16 mm; greatest width (EW) 1.12 – 1.15 mm; dorsal side dark brown with greenish iridescence; venter brown to almost black, tarsal claws reddish-brown. Dorsal surface densely covered with short recumbent setae and sparser, longer, dark, semi-erect setae; ventral surface densely covered with longer, golden, recumbent setae, especially on trochanters.

Head partly retractable into prothorax. Clypeus with anterior margin straight, about three times wider than long, shorter and narrower than labrum. Labrum feebly emarginate anteromedially, expanded laterally with sides broadly rounded, densely setose. Frontoclypeal suture visible, almost straight. Eyes suboval in lateral view, protruding from head outline, bordered by long black curved setae (“eyelashes”) that arise near dorsal and ventral sides of eyes and extend toward middle of eye. Antenna moniliform, 11-segmented, pubescent; first two segments with dense long, dark brown setae, rest of antenna with only few such setae on sides; scape curved, about twice as long as pedicel, remaining segments about three times longer than first and second combined; segments 3–10 short, subtriangular; terminal segment subglobular with slightly pointed apex.

Pronotum (PL) 0.69 – 0.77 mm long, widest (PW: 0.88 – 0.92 mm) at base; with complete transversal depression at apical third and small basolateral impressions, with two prescutellar foveae; sublateral carinae absent; lateral margins convex before and after depression, basal angles slightly projected outwards; disc raised with concave sides near base; two tiny depressed dots medially near base; middle portion of base produced posteriorly; basal margin straight on sides, broadly rounded before scutellum. Scutellum subtriangular. Hypomeron narrow, straight. Prosternum extremely short in front of procoxae; prosternal process parallel-sided, apical portion subtriangular. Mesoventrite short with a deep, broad, V-shaped depression for reception of prosternal process. Metaventrite long and wide, slightly depressed along midline; discrimen thin and long, reaching abdomen. Legs slender, long. Procoxae and mesocoxae rounded, metacoxae transverse. Forelegs shortest, with all segments slightly wider than remaining pairs. Mesotibiae with medial pubescent area long, reaching before apex and lateral pubescent area long, extending to 2/3 of tibia. Mesotibiae with short thorn-like carina on inner apex, metatibiae with feeble thorn-like carina on inner apex. Tarsi simple, fourth tarsal segment with fine, nearly erect setae ventrally, fifth segment longest. Tarsal claws long and stout.

Elytra (EL) 2.28 – 2.42 mm long, widest (EW: 1.12 – 1.15 mm) across humeri; subparallel in anterior 4/5, with ten rows of small punctures forming striae; punctures separated by a distance three to four times the puncture diameter; humeral area slightly swollen. First four or five striae distinct, in nearly straight lines, remaining ones feebly visible, obscured apically. Epipleuron thin, widest in anterior third. Apical margin of elytra narrowly rounded.

Abdomen with five clearly visible ventrites (Fig. 12). Intercoxal process subtriangular with rounded apex. First three ventrites depressed medially; fifth ventrite deeply and broadly emarginate. Cuticle densely covered with short, golden, recumbent setae. Aedeagus (Figs 23, 24) elongate. Penis in ventral view narrowing from short basal apophyses towards rounded apex with right margin slightly dilated in middle, in lateral view slender, sinuate, with widened basal fourth; with corona membranous, fibula not visible, curved oblong sclerotized structure present in middle. Parameres slightly longer than half of penis, in lateral view widest at base, moderately tapering towards rounded apex, in ventral view jointed in middle, with rounded apex. Phallobase parallel-sided, curved in lateral view. Penis and parameres with sparse fine spines.

**Figures 6–11.**
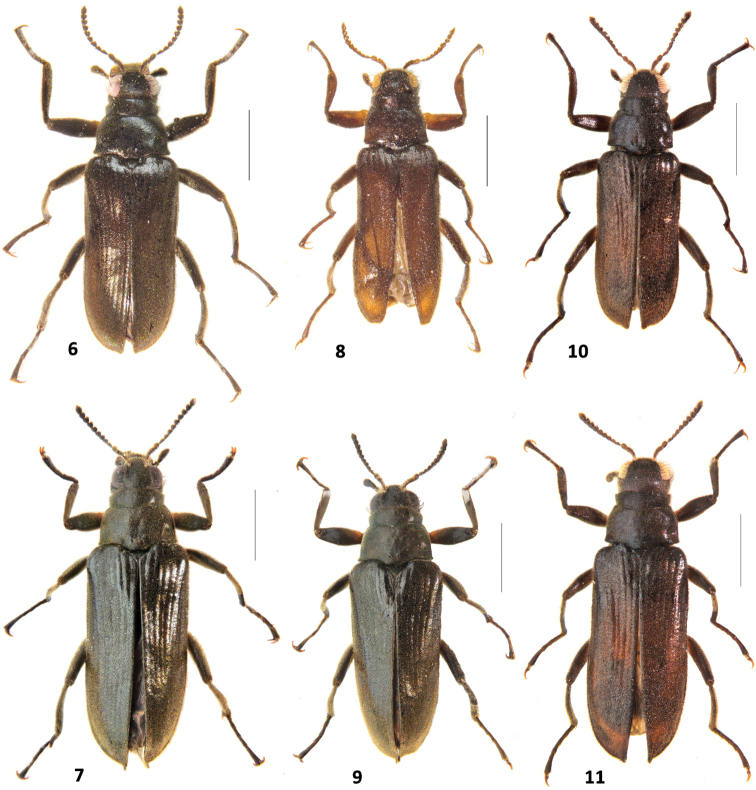
*Hexanchorushabitus*: **6***H.onoreionorei* sp. n. male **7***H.onoreionorei* sp. n. female **8***H.onoreisagittatus* ssp. n. male **9***H.onoreisagittatus* ssp. n. female **10***H.shepardi* sp. n. male **11***H.shepardi* sp. n. female. Scale: 1 mm.

**Figures 12–22.**
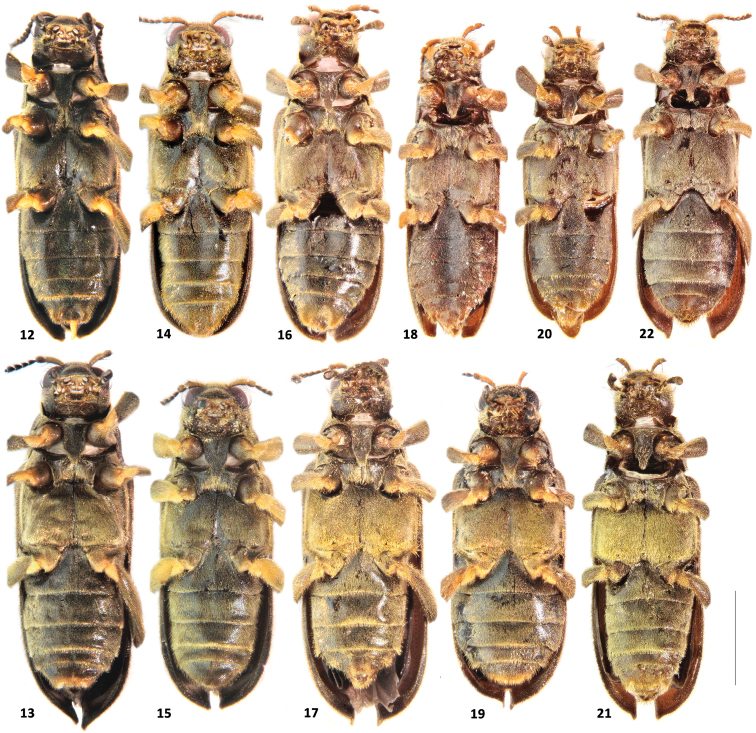
*Hexanchorus* ventral view: **12***H.cordillierae* male **13***H.cordillierae* female **14**) *H.virilis* sp. n. male **15**H.cfvirilis sp. n. female **16***H.onoreionorei* sp. n. male **17***H.onoreionorei* sp. n. female **18***H.onoreisagittatus* ssp. n. male **19***H.onoreisagittatus* ssp. n. female **20***H.shepardi* sp. n. male **21***H.shepardi* sp. n. female **22***H.rostratus* sp. n. male. Scale: 1 mm.

**Female.** Externally similar to male (Figs 2, 13) except bigger; elytra broader with slightly produced apex; meso – and metatibiae without carina on inner apex; first three ventrites medially convex and fifth ventrite very broadly but shallowly emarginate. Females vary in size (CL: 3.25 – 3.36 mm, PL: 0.70 – 0.71 mm, PW: 0.86 – 0.95 mm, EL: 2.54 – 2.66 mm, EW: 1.14 – 1.26 mm).

**Variation.** We observed variation in color from dark brown to brown, size and pubescence, especially on abdominal sterna. Scale of green iridescence differed substantially.

#### Distribution.

Until now, the species was known only from Colombia. We recorded *H.cordillierae* at two localities in the Napo Province and three localities in Pastaza Province (Fig. 36). This is the first record of *H.cordillierae* for Ecuador.

#### Note.

We had habitus and aedaeagus photographs of the type available in this study, and were kindly provided with a redescription by Cinzia Monte, which was made based on the study of the type specimen. Based on the comparison of our specimens with the redescription of *H.cordillierae*, we have assigned the studied specimens to *H.cordillierae*.

### Hexanchorus virilis sp. n.

Taxon classificationAnimaliaColeopteraElmidae

http://zoobank.org/E4223A38-3093-4EB0-B4EF-C07705D555A0

Figs 3 , 4 , 14 , 15 , 25 , 26 , 36

#### Material examined.

**Holotype** (PUCE) ♂: “Ecuador, Pastaza prov., Río Uklan, 01°17’13.8”S, 77°38’52.5”W 468m a.s.l., 18.8.2013, bigger river with lowland character, stream ca 15m wide, slow flowing with small riffles, with boulders, rock tables and sand, Čiampor & Čiamporová-Zaťovičová lgt.”. **Paratypes** (PUCE, NMW, CCB): 10 ♂♂ with the same locality as holotype.

#### Diagnosis.

*Hexanchorusvirilis* sp. n. can be distinguished from all species of the genus by combination of the following male characters: 1) smaller size (CL: 2.78 – 2.97 mm); 2) protibiae apically dilated; 3) mesotibiae with medial pubescent area long, reaching to 2/4 of tibia and lateral pubescent area short, only in first fourth; 4) mesotibiae with small tubercle on inner apex; 5) metatibiae with indistinct tubercle on inner apex; 6) elytra with rounded apices; 7) fifth ventrite moderately deeply but narrowly emarginate; 8) aedeagus with slightly zagged apical portion in ventral view.

#### Description.

**Male.** Body elongate, subparallel, dorsum moderately convex (Fig. 3). Length (CL) 2.78 – 2.97 mm; greatest width (EW) 1.02 – 1.07 mm, dorsal side dark brown with greenish iridescence; venter brown to almost black, tarsal claws reddish-brown. Dorsal surface densely covered with short recumbent setae and sparser, longer, dark, semi-erect setae; ventral surface densely covered with longer, golden, recumbent setae, especially on trochanters.

Head partly retractable into prothorax. Clypeus with anterior margin straight, about three times wider than long, shorter and narrower than labrum. Labrum feebly emarginate anteromedially, expanded laterally with sides broadly rounded, densely setose. Frontoclypeal suture visible, almost straight. Eyes suboval in lateral view, protruding from head outline, bordered by long black curved setae (“eyelashes”) that arise near dorsal and ventral sides of eyes and extend toward middle of eye. Antenna moniliform, 11-segmented, pubescent; first two segments with dense long, dark brown setae, rest of antenna with only few such setae on sides; scape curved, about twice as long as pedicel, remaining segments about three times longer than first and second combined; segments 3–10 short, subtriangular; terminal segment subglobular with slightly pointed apex. Pronotum (PL) 0.65 – 0.69 mm long, widest (PW: 0.81 – 0.83 mm) at base; with complete transversal depression at apical third and small basolateral impressions, with two prescutelar foveae; sublateral carinae absent; lateral margins convex before and after depression, basal angles slightly projected outwards; disc raised with concave sides near base; two tiny depressed dots medially near base; middle portion of base produced posteriorly; basal margin straight on sides, broadly rounded before scutellum. Scutellum subtriangular. Hypomeron narrow, straight. Prosternum extremely short in front of procoxae; prosternal process parallel-sided, apical portion subtriangular. Mesoventrite short with a deep, broad, V-shaped depression for reception of prosternal process. Metaventrite long and wide, slightly depressed along midline; discrimen thin and long, reaching abdomen. Legs slender, long. Procoxae and mesocoxae rounded, metacoxae transverse. Forelegs shortest, with all segments slightly wider than remaining pairs. Protibiae apically widened, emarginated before apex. Mesotibiae with medial pubescent area long, reaching to 2/4 of tibia and lateral pubescent area short, only in first fourth. Mesotibiae with small tubercle on inner apex, metatibiae with small tubercle on inner apex. Tarsi simple, fourth tarsal segment with fine, nearly erect setae ventrally, fifth segment longest. Tarsal claws long and stout.

Elytra (EL) 1.91 – 2.16 mm long, widest (EW: 1.02 – 1.07 mm) across humeri; subparallel in anterior 4/5, with ten rows of small punctures forming striae; punctures separated by a distance three to four times the puncture diameter; humeral area slightly swollen. First four or five striae distinct, in nearly straight lines, remaining ones feebly visible, obscured apically. Epipleuron thin, widest in anterior third. Apical margin of elytra narrowly rounded.

Abdomen with five clearly visible ventrites (Fig. 14). Intercoxal process subtriangular with rounded apex. First three ventrites depressed medially; fifth ventrite moderately deeply but narrowly emarginate. Cuticle densely covered with short, golden, recumbent setae. Aedeagus (Figs 25, 26) elongate. Penis in ventral view subparallel with long apophyses, apical part slightly zagged, firstly wide then strongly narrowing into long apical portion with rounded apex, in lateral view slender, sinuate, with widened basal third; with corona membranous, fibula not visible, curved oblong sclerotized structure present in middle. Parameres asymmetrical, about 1.5x shorter than penis, in lateral view subparallel, widest in middle, feebly tapering towards rounded apex, skewed on one side, in ventral view jointed in middle, with rounded apex. Phallobase parallel-sided, feebly curved in lateral view. Penis and parameres with sparse fine spines.

**Figures 23–26.**
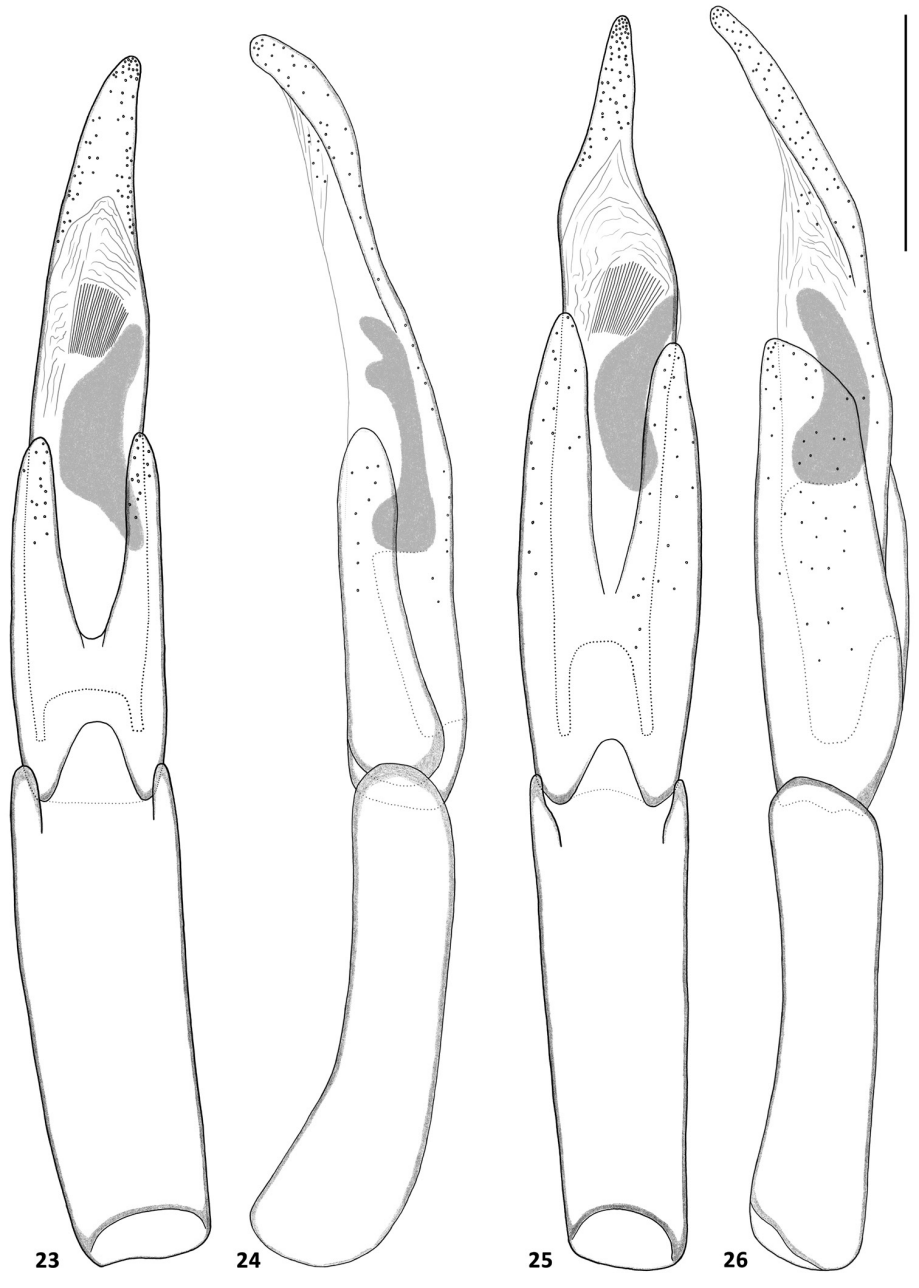
Aedeagi of *Hexanchorus*: **23***H.cordillierae* ventral view **24***H.cordillierae* lateral view **25***H.virilis* sp. n. ventral view **26***H.virilis* sp. n. lateral view. Scale: 0.1 mm.

**Female.** Even females were collected at the same locality as males, we failed to get molecular data from them to confirm their conspecificity. Due to that we refrained from formal description of females and including them in the type series, but we provide their habitus photographs (Figs 4, 15).

**Variation.** We observed variation in size, color from dark brown to brown and pubescence, especially on abdominal sterna. Scale of green iridescence differed substantially.

#### Etymology.

Latin, *virilis* (manly, masculine, virile), in reference to male sexual dimorphism.

#### Distribution.

Known only from the one locality in Pastaza Province (Fig. 36).

### Hexanchorus rostratus sp. n.

Taxon classificationAnimaliaColeopteraElmidae

http://zoobank.org/FCBFC399-3D18-45D2-B0A8-5CEA797CD5DB

**Figures 27–30.**
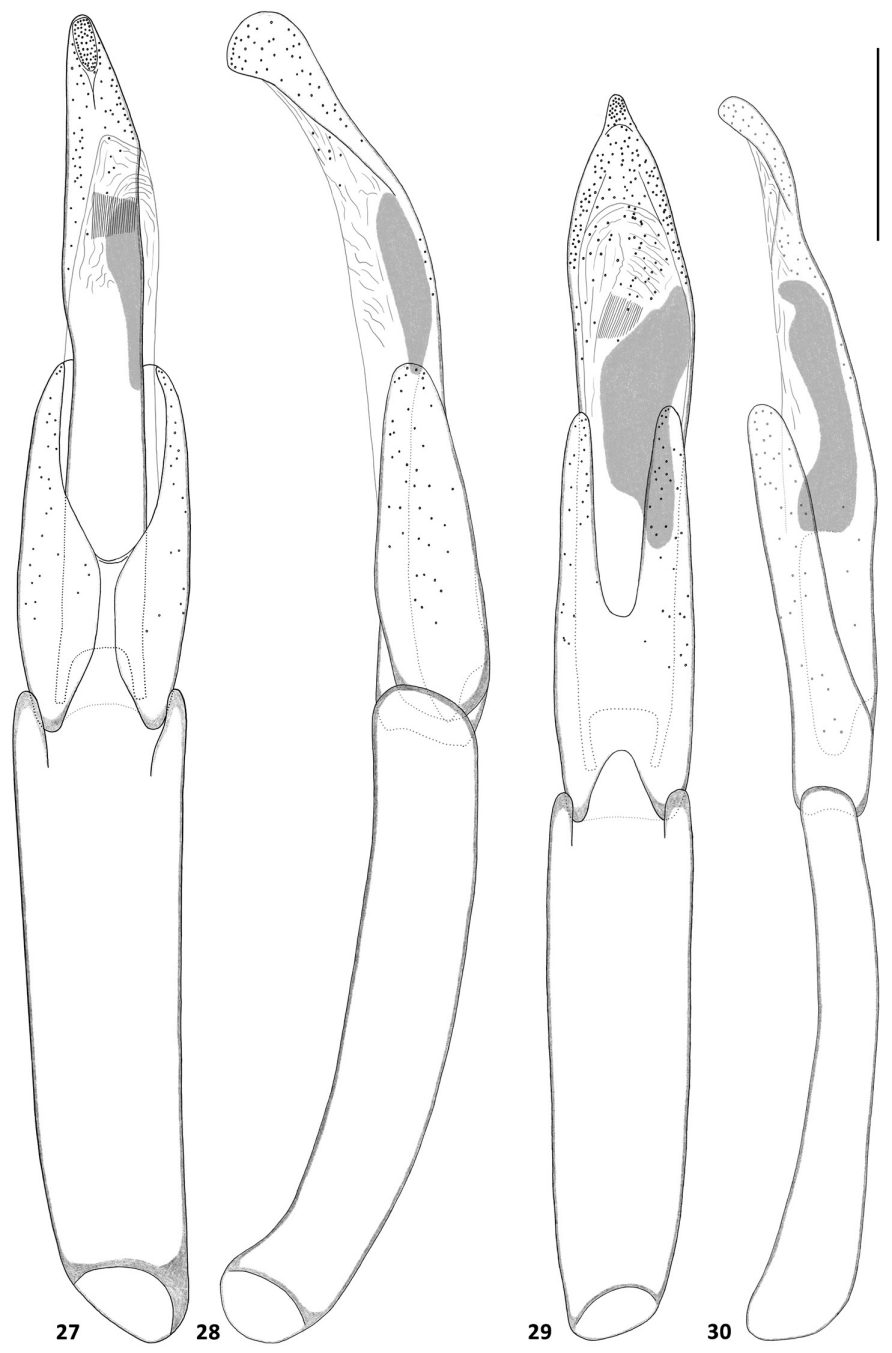
Aedeagi of *Hexanchorus*: **27***H.rostratus* sp. n. ventral view **28***H.rostratus* sp. n. lateral view **29***H.shepardi* sp. n. ventral view **30**) *H.shepardi* sp. n. lateral view. Scale: 0.1 mm.

Figs 5 , 22 , 27 , 28 , 36

#### Material examined.

**Holotype** (PUCE) ♂: “Ecuador, MoronaSantiago prov., Limón env., Río Yungantza, 02°59’49.3”S, 78°29’18.9”W 1522m a.s.l., 27.8.2013, stream ca 3m wide, fast flowing, partly shaded, with boulders, stones, gravel, Čiampor & Čiamporová-Zaťovičová lgt.”. **Paratypes** (PUCE): 2 ♂♂ with the same data as holotype.

#### Diagnosis.

*Hexanchorusrostratus* sp. n. can be distinguished from all species of the genus by combination of the following male characters: 1) bigger size (CL: 3.46 – 3.58 mm); 2) mesotibiae with medial pubescent area extremely short, only at base and lateral pubescent area short, reaching to 1/4 of tibia 3) mesotibiae with indistinct tubercle on inner apex; 4) metatibiae with indistinct tubercle on inner apex; 5) elytra with slightly acute, almost rounded apices; 6) fifth ventrite moderately deeply but narrowly emarginate; 7) aedeagus with beak-like apical portion in lateral view.

#### Description.

**Male.** Body elongate, subparallel, dorsum moderately convex (Fig. 5). Length (CL) 3.46 – 3.58 mm; greatest width (EW) 1.25 – 1.32 mm, dorsal side brown with greenish iridescence; venter brown to almost black, tarsal claws reddish-brown. Dorsal surface densely covered with short recumbent setae and sparser, longer, dark, semi-erect setae; ventral surface densely covered with longer, golden, recumbent setae, especially on trochanters.

Head partly retractable into prothorax. Clypeus with anterior margin straight, about three times wider than long, shorter and narrower than labrum. Labrum feebly emarginate anteromedially, expanded laterally with sides broadly rounded, densely setose. Frontoclypeal suture visible, almost straight. Eyes suboval in lateral view, protruding from head outline, bordered by long black curved setae (“eyelashes”) that arise near dorsal and ventral sides of eyes and extend toward middle of eye. Antenna moniliform, 11-segmented, pubescent; first two segments with dense long, dark brown setae, rest of antenna with only few such setae on sides; scape curved, about twice as long as pedicel, remaining segments about three times longer than first and second combined; segments 3–10 short, subtriangular; terminal segment subglobular with slightly pointed apex.

Pronotum (PL) 0.77 – 0.85 mm long, widest (PW: 0.96 – 1.03 mm) at base; with complete transversal depression at apical third and small basolateral impressions, with two prescutelar foveae; sublateral carinae absent; lateral margins convex before and after depression, basal angles slightly projected outwards; disc raised with concave sides near base; two tiny depressed dots medially near base; middle portion of base produced posteriorly; basal margin straight on sides, broadly rounded before scutellum. Scutellum subtriangular. Hypomeron narrow, straight. Prosternum extremely short in front of procoxae; prosternal process parallel-sided, apical portion subtriangular. Mesoventrite short with a deep, broad, V-shaped depression for reception of prosternal process. Metaventrite long and wide, slightly depressed along midline; discrimen thin and long, reaching abdomen. Legs slender,long. Procoxae and mesocoxae rounded, metacoxae transverse. Forelegs shortest, with all segments slightly wider than remaining pairs. Mesotibiae with medial pubescent area extremely short, only at base and lateral pubescent area short, reaching to 1/4 of tibia. Mesotibiae and metatibiae with indistinct tubercle on inner apex. Tarsi simple, fourth tarsal segment with fine, nearly erect setae ventrally, fifth segment longest. Tarsal claws long and stout.

Elytra (EL) 2.69 – 2.73 mm long, widest (EW: 1.25 – 1.32 mm) across humeri; with ten rows of small punctures forming striae; punctures separated by a distance three to four times the puncture diameter; humeral area slightly swollen. First four or five striae distinct, in nearly straight lines, remaining ones feebly visible, obscured apically. Epipleuron thin, widest in anterior third. Apical margin of elytra acutely produced.

Abdomen with five clearly visible ventrites (Fig. 22). Intercoxal process subtriangular with rounded apex. First three ventrites depressed medially; fifth ventrite moderately deeply but narrowly emarginate. Cuticle densely covered with short, golden, recumbent setae. Aedeagus (Figs 27, 28). elongate. Penis in ventral subparallel with distinct apophyses, narrowest in middle, with rounded apex, in lateral with subglobular apex skewed from below, strongly constricted then widened in basal half; with corona membranous, fibula not visible, straight oblong sclerotized structure present in apical half. Parameres about half as long as penis, in lateral view widest in basal half, tapering towards rounded apex, in ventral view with thin rounded apex, distinctly widening in apical half; Phallobase long, parallel-sided, curved in lateral view. Penis and parameres with sparse fine spines.

**Female.** Unknown.

**Variation.** We observed variation in size and pubescence, especially on abdominal sterna. Scale of green iridescence differed substantially.

#### Etymology.

Latin, *rostrātus* (beak-shaped), in reference to the apical part of penis in lateral view that resembles an upper beak of some birds.

#### Distribution.

Known only from the one locality in Morona-Santiago Province (Fig. 36).

### Hexanchorus onorei onorei ssp. n.

Taxon classificationAnimaliaColeopteraElmidae

http://zoobank.org/BF0BB8DA-D2DB-4C16-ABEE-F46AD8648913

**Figures 31–34.**
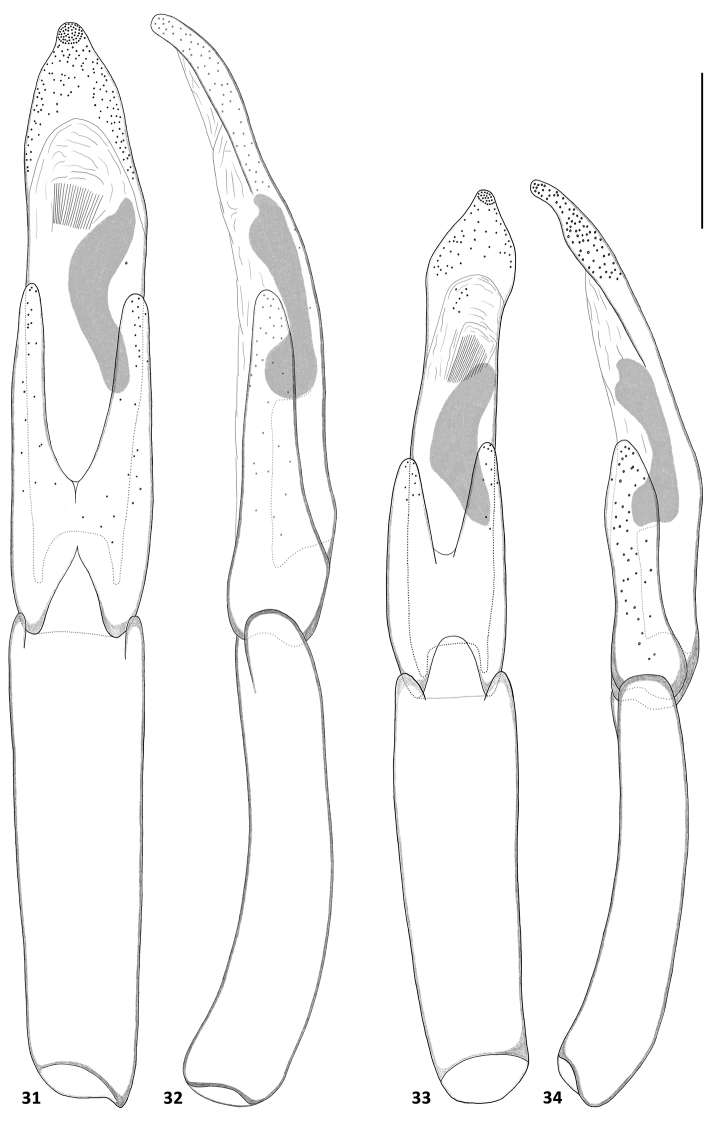
Aedeagi of *Hexanchorus*: **31***H.onoreionorei* sp. n. ventral view **32***H.onoreionorei* sp. n. lateral view **33***H.onoreisagittatus* ssp. n. ventral view **34***H.onoreisagittatus* ssp. n. lateral view. Scale: 0.1 mm.

Figs 6 , 7 , 16 , 17 , 31 , 32 , 36

#### Material examined.

**Holotype** (PUCE) ♂: “Ecuador, Morona-Santiago prov., Indanza env., Río Crusado, 03°02’55.0”S, 78°30’03.5”W 972m a.s.l., 24.8.2013, stream ca 5m wide, fast flowing with rapids, in forest, with gravel, boulders, Čiampor & Čiamporová-Zaťovičová lgt.” **Paratypes** (PUCE, NMW, CCB): 1 ♂, 2 ♀♀ with the same data as holotype; 2 ♂♂ “Ecuador, Morona-Santiago prov., Río Indanza, Indanza env., 03°04‘09.3“S, 78°28‘07.9“W 772m a.s.l., 28.8.2013, at light, Čiampor & Čiamporová-Zaťovičová lgt.”.

#### Diagnosis.

*Hexanchorusonoreionorei* sp. n. can be distinguished from all species of the genus by combination of the following male characters: 1) bigger size (CL: 3.44 – 3.57 mm); 2) mesotibiae with medial pubescent area through entire tibia and lateral pubescent area reaching half of tibia; 3) mesotibiae with small tubercle on inner apex; 4) metatibiae with indistinct tubercle on inner apex; 5) elytra with slightly acute, almost rounded apices; 6) fifth ventrite deeply and broadly emarginate; 7) aedeagus broad with protruded apex in ventral view.

#### Description.

**Male.** Body elongate, subparallel, dorsum moderately convex (Fig. 6). Length (CL) 3.44 – 3.57 mm; greatest width (EW) 1.28 – 1.36 mm, dorsal side dark brown with greenish iridescence; venter brown to almost black, tarsal claws reddish-brown. Dorsal surface densely covered with short recumbent setae and sparser, longer, dark, semi-erect setae; ventral surface densely covered with longer, golden, recumbent setae, especially on trochanters.

Head partly retractable into prothorax. Clypeus with anterior margin straight, about three times wider than long, shorter and narrower than labrum. Labrum feebly emarginated anteromedially, expanded laterally with sides broadly rounded, densely setose. Frontoclypeal suture visible, almost straight. Eyes suboval in lateral view, protruding from head outline, bordered by long black curved setae (“eyelashes”) that arise near dorsal and ventral sides of eyes and extend toward middle of eye. Antenna moniliform, 11-segmented, pubescent; first two segments with dense long, dark brown setae, rest of antenna with only few such setae on sides; scape curved, about twice as long as pedicel, remaining segments about three times longer than first and second combined; segments 3–10 short, subtriangular; terminal segment subglobular with slightly pointed apex.

Pronotum (PL) 0.82 – 0.85 mm long, widest (PW: 1.04 – 1.09 mm) at base; with complete transversal depression at apical third and small basolateral impressions, with two prescutelar foveae; sublateral carinae absent; lateral margins convex before and after depression, basal angles slightly projected outwards; disc raised with concave sides near base; two tiny depressed dots medially near base; middle portion of base produced posteriorly; basal margin straight on sides, broadly rounded before scutellum. Scutellum subtriangular. Hypomeron narrow, straight. Prosternum extremely short in front of procoxae; prosternal process parallel-sided, apical portion subtriangular. Mesoventrite short with a deep, broad, V-shaped depression for reception of prosternal process. Metaventrite long and wideslightly depressed along midline; discrimen thin and long, reaching abdomen. Legs slender, long. Procoxae and mesocoxae rounded, metacoxae transverse. Forelegs shortest, with all segments slightly wider than remaining pairs. Mesotibiae with medial pubescent area through entire tibia and lateral pubescent area reaching to half. Mesotibiae with small tubercle on inner apex, metatibiae with indistinct tubercle on inner apex. Tarsi simple, fourth tarsal segment with fine, nearly erect setae ventrally, fifth segment longest. Tarsal claws long and stout.

Elytra (EL) 2.63 – 2.72 mm long, widest (EW: 1.28 – 1.36 mm) across humeri; subparallel in anterior 4/5, with ten rows of small punctures forming striae; punctures separated by a distance three to four times the puncture diameter; humeral area slightly swollen. First four or five striae distinct, in nearly straight lines, remaining ones feebly visible, obscured apically. Epipleuron thin, widest in anterior third. Apical margin of elytra narrowly rounded.

Abdomen with five clearly visible ventrites (Fig. 16). Intercoxal process subtriangular with rounded apex. First three ventrites depressed medially; fifth ventrite deeply and broadly emarginate. Cuticle densely covered with short, golden, recumbent setae. Aedeagus (Figs 31, 32) elongate. Penis in ventral view subparallel with short apophyses, apical part narrowing towards protruded rounded apex, in lateral view slender, sinuate, with widened basal third, with corona membranous, fibula not visible, curved oblong sclerotized structure present in middle. Parameres about 1.5 times shorter than penis, in lateral view widest at base, tapering towards rounded apex, in ventral view jointed in middle, with rounded apex. Phallobase parallel-sided, curved in lateral view. Penis and parameres with fine, sparse spines.

**Female.** Externally similar to male (Figs 7, 17) except bigger (CL: 3.83 – 3.88 mm); elytra with pointed and vertically curved apices; meso – and metatibiae without carina on inner apex; first three ventrites medially convex and fifth ventrite only feebly emarginate. Females vary in size (PL: 0.75 – 0.77 mm, PW: 1.10 – 1.12 mm, EL: 3.07 – 3.11 mm, EW: 1.40 – 1.43 mm).

**Variation.** We observed variation in color from dark brown to brown, size and pubescence, especially on abdominal sterna. Scale of green iridescence differed substantially.

#### Etymology.

This species is named after our friend, Prof. Giovanni Onore, President of the Otonga Foundation, to express our gratitude for his altruistic help and support for research of Elmidae fauna of Ecuador.

#### Distribution.

Known from the two localities in Morona-Santiago Province (Fig. 36).

### Hexanchorus onorei sagittatus ssp. n.

Taxon classificationAnimaliaColeopteraElmidae

http://zoobank.org/EEDA9052-48D5-49C7-909A-854C020F9765

Figs 8 , 9 , 18 , 19 , 33 , 34 , 36

#### Material examined.

**Holotype** (PUCE) ♂: “Ecuador, Morona-Santiago prov., Río Indanza, Indanza env., 03°04‘09.3“S, 78°28‘07.9“W 772m a.s.l., 28.8.2013, at light, Čiampor & Čiamporová-Zaťovičová lgt.”. **Paratypes** (PUCE, NMW, CCB): 1 ♂, 5 ♀♀ with the same data as holotype.

#### Diagnosis.

*Hexanchorusonoreisagittatus* ssp. n. (Figs 8, 18) is externally similar to *Hexanchorusonoreionorei* sp. n. but can be distinguished by combination of the following male characters: 1) smaller size (CL: 3.22 – 3.25 mm vs 3.44 – 3.57 mm); 2) fifth ventrite distinctly wider emarginate; 3) aedeagus arrow-like in ventral view.

#### Description.

**Male.** Aedeagus (Figs 33, 34) elongate. Penis in ventral view arrow-like, mostly subparallel with short apophyse, tapering towards apex, then widened into subglobular portion with protruded, rounded apex, in lateral view with apex emarginate, then slender, sinuate, with widened basal third, with corona membranous, fibula not visible, curved oblong sclerotized structure present in middle. Parameres slightly asymmetrical, about half as long as penis, in lateral view widest at base, narrowest in middle, apical part with rounded apex, in ventral view jointed in middle, with rounded apex. Phallobase parallel-sided, curved in lateral view. Penis and parameres with sparse fine spines.

**Figure 35.**
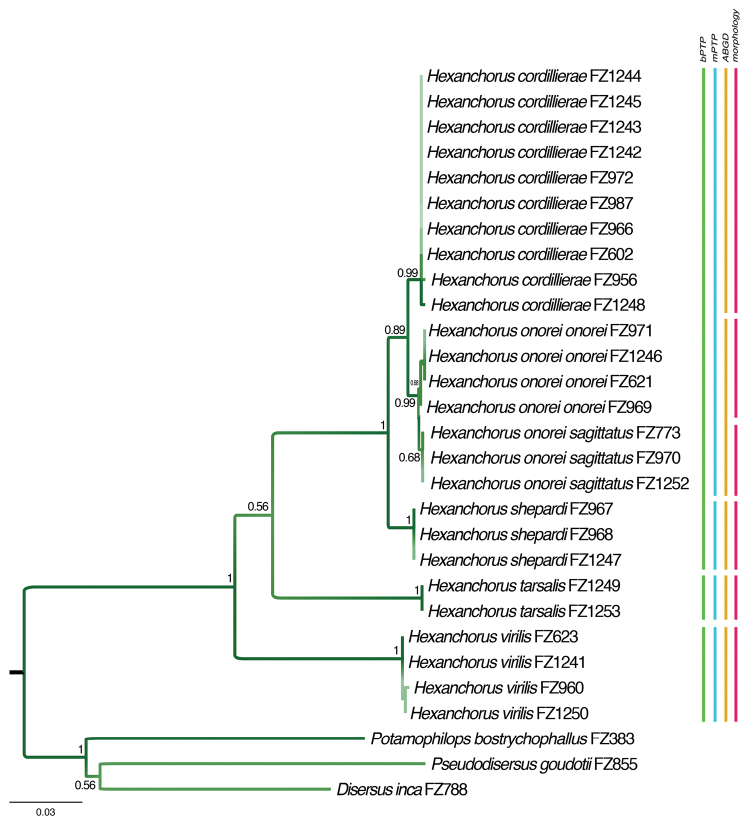
Maximum likelihood analysis tree based on 625 bp barcoding fragment of COI with species delimitation according to different approaches used. Numbers next to branches represent Bootstrap support (ML).

**Female.** Externally similar to male (Figs 9, 19) except bigger (CL: 3.65 – 3.68 mm); elytra with pointed and vertically curved apices; meso – and metatibiae without carina on inner apex; first three ventrites medially convex and fifth ventrite only feebly emarginated. Females vary in size (PL: PL: 0.76 – 0.78 mm, PW: 1.07 – 1.09 mm, EL: 2.88 – 2.90 mm, EW: 1.35 – 1.37 mm).

**Variation.** We observed variation in size and pubescence, especially on abdominal sterna. Scale of green iridescence differed substantially.

#### Etymology.

Latin, *sagittātus* (formed like arrow), in reference to its arrow-like shape of penis.

#### Distribution.

**Figure 36.**
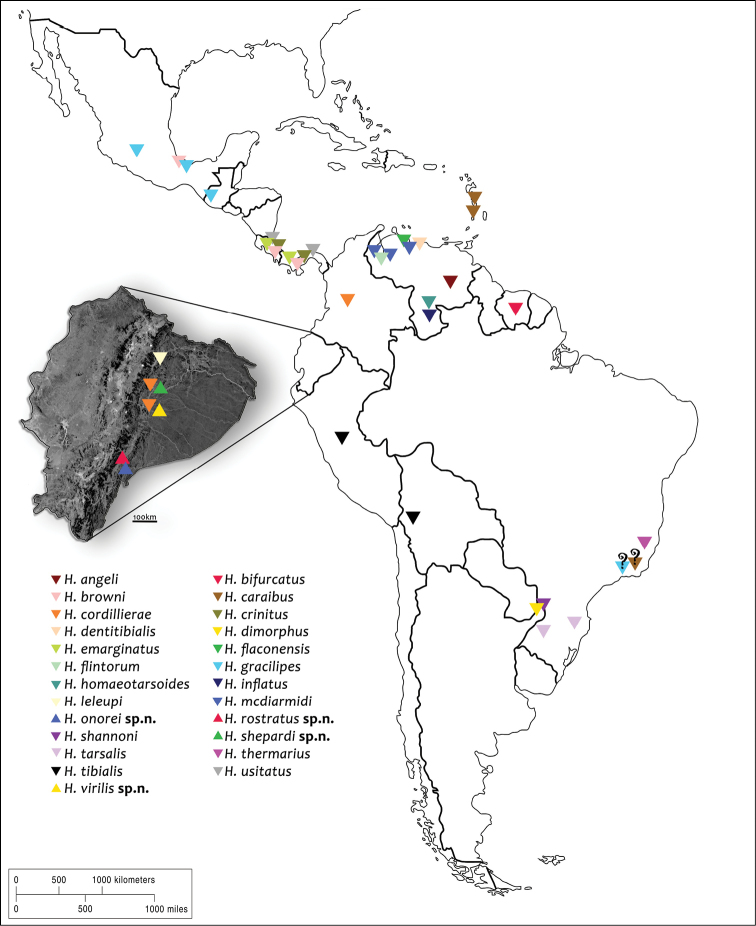
Distribution map of all known *Hexanchorus* species. (top down triangle – known species, top up triangle – new species, question mark – doubtful distribution).

Known only from the one locality in Morona-Santiago Province (Fig. 36).

### Hexanchorus shepardi sp. n.

Taxon classificationAnimaliaColeopteraElmidae

http://zoobank.org/F8C4E2E8-15F5-4588-B249-F68DF2FD2A0D

Figs 10 , 11 , 20 , 21 , 29 , 30 , 36

#### Material examined.

**Holotype** (PUCE) ♂: “Ecuador, Napo prov., road to Coca, Sumaco env., 00°42’25.7”S, 77°43’10.0”W 1138m a.s.l., 17.8.2013, stream ca 2–3 m wide, fast flowing, with boulders, stones, gravel, submerged wood, Čiampor & Čiamporová-Zaťovičová lgt.” **Paratypes** (PUCE, NMW, CCB): 1 ♂, 5 ♀♀ with the same data as holotype.

#### Diagnosis.

*Hexanchorusshepardi* sp. n. can be distinguished from all species of the genus by combination of the following male characters: 1) moderate size (CL: 3.22 – 3.36 mm); 2) mesotibiae with medial pubescent area long, extending before apex and lateral pubescent area shorter reaching behind first third; 3) mesotibiae with small tubercle on inner apex; 4) metatibiae with indistinct tubercle on inner apex; 5) elytra with slightly acute, almost rounded apices; 6) fifth ventrite deeply and broadly emarginate; 7) aedeagus with ovate apical portion in ventral view.

#### Description.

**Male.** Body elongate, subparallel, dorsum moderately convex (Fig. 10). Length (CL) 3.22 – 3.36 mm; greatest width (EW) 1.31 – 1.37 mm, dorsal side dark brown with greenish iridescence; venter brown to almost black, tarsal claws reddish-brown. Dorsal surface densely covered with short recumbent setae and sparser, longer, dark, semi-erect setae; ventral surface densely covered with longer, golden, recumbent setae, especially on trochanters.

Head partly retractable into prothorax. Clypeus with anterior margin straight, about three times wider than long, shorter and narrower than labrum. Labrum feebly emarginated anteromedially, expanded laterally with sides broadly rounded, densely setose. Frontoclypeal suture visible, almost straight. Eyes suboval in lateral view, protruding from head outline, bordered by long black curved setae (“eyelashes”) that arise near dorsal and ventral sides of eyes and extend toward middle of eye. Antenna moniliform, 11-segmented, pubescent; first two segments with dense long, dark brown setae, rest of antenna with only few such setae on sides; scape curved, about twice as long as pedicel, remaining segments about three times longer than first and second combined; segments 3–10 short, subtriangular; terminal segment subglobular with slightly pointed apex.

Pronotum (PL) 0.83 – 0.87 mm long, widest (PW: 1.07 – 1.08 mm) at base; with complete transversal depression at apical third and small basolateral impressions, with two prescutelar foveae; sublateral carinae absent; lateral margins convex before and after depression, basal angles slightly projected outwards; disc raised with concave sides near base; two tiny depressed dots medially near base; middle portion of base produced posteriorly; basal margin straight on sides, broadly rounded before scutellum. Scutellum subtriangular. Hypomeron narrow, straight. Prosternum extremely short in front of procoxae; prosternal process parallel-sided, apical portion subtriangular. Mesoventrite short with a deep, broad, V-shaped depression for reception of prosternal process. Metaventrite long and wide, slightly depressed along midline; discrimen thin and long, reaching abdomen. Legs slender, long. Procoxae and mesocoxae rounded, metacoxae transverse. Forelegs shortest, with all segments slightly wider than remaining pairs. Mesotibiae with medial pubescent area long, extending before apex and lateral pubescent area shorter reaching behind first third. Mesotibiae with small tubercle on inner apex, metatibiae with indistinct tubercle on inner apex. Tarsi simple, fourth tarsal segment with fine, nearly erect setae ventrally, fifth segment longest. Tarsal claws long and stout.

Elytra (EL) 2.42 – 2.63 mm long, widest (EW: 1.31 – 1.37 mm) across humeri; subparallel in anterior 4/5, with ten rows of small punctures forming striae; punctures separated by a distance three to four times the puncture diameter; humeral area slightly swollen. First four or five striae distinct, in nearly straight lines, remaining ones feebly visible, obscured apically. Epipleuron thin, widest in anterior third. Apical margin of elytra acutely produced.

Abdomen with five clearly visible ventrites (Fig. 20). Intercoxal process subtriangular with rounded apex. First three ventrites depressed medially; fifth ventrite deeply and broadly emarginate. Cuticle densely covered with short, golden, recumbent setae. Aedeagus (Figs 29, 30) elongate. Penis in ventral view subparallel with short apophyses, apical part ovate with rounded apex, in lateral view slender, sinuate, with widened basal third, with corona membranous, fibula not visible, curved oblong sclerotized structure present in middle. Parameres about 1.7x shorter than penis, in lateral view widest at base, moderately tapering towards rounded apex, in ventral view jointed in middle, with rounded apex. Phallobase parallel-sided, in later view curved and slender. Penis and parameres with sparse fine spines.

**Female.** Externally similar to male (Figs 11, 21) except bigger (CL: 3.58 – 3.62 mm); meso – and metatibiae without carina on inner apex; first three ventrites medially convex and fifth ventrite very broadly but shallowly emarginate. Females vary only slightly in size (PL: 0.77 – 0.78 mm, PW: 1.00 – 1.01 mm, EL: 2.81 – 2.84 mm, EW: 1.24 – 1.27 mm).

**Variation.** We observed variation in size and in pubescence, especially on abdominal sterna, was observed. Scale of green iridescence differed substantially.

#### Etymology.

The species is named after Prof. William D. Shepard, great American coleopterologist and expert on dryopoid beetles.

#### Distribution.

Known only from the one locality in Napo Province (Fig. 36).

## Discussion

Ecuador is one of the richest countries in the world, in regard to its biodiversity. Here we focused on the riffle beetle genus *Hexanchorus*. Although we analysed the material from a relatively small area, the results clearly demonstrate that the diversity of the genus is almost certainly much higher than it would appear based on the previous knowledge. With its 25 species, *Hexanchorus* is the most diverse Larainae genus in Latin America, forming an important part of the Elmidae fauna in the region.

Most of the *Hexanchorus* species are very similar concerning their external morphology and usually it is very hard to identify species without examining male genitalia. Moreover, different species sometimes inhabit the same stream or are collected together at light, which makes assigning females to the species difficult. Hence we also employed molecular data (DNA barcoding), which has proved very useful for Elmidae taxonomy in previous studies (e.g. Čiampor Jr et al. 2013, 2016, 2017) and allowed for the inclusion of females in the type series in most of the described species.

The description of subspecies in Elmidae genera is usually based on subtle morphological differences between geographically isolated populations (e.g. Jeng and Yang 1993; Jäch 1994). The two subspecies of *H.onorei* sp. n. were collected at the same locality. They clearly differ in the morphology of the male genitalia, but due to the small genetic distance they most probably represent separate lineages of the same species occuring sympatrically after a short period of isolation. However, subspecies designation could be useful not only for allopatric populations, but also in situations where secondary contact between distinct populations has occurred (Monroe 1982). The latter could be the case of the subspecies of *H.onorei*.

Regarding Ecuador, only *H.leleupi* Delève, 1968 was known from this country until now (Monte and Mascagni 2012). This species was collected at high altitudes (3300 m a.s.l.), which contrasts with other species occurring mostly up to 1500 m a.s.l. (Spangler and Santiago-Fragoso 1992 and our own observation).

Based on the limited literature sources (Guérin-Méneville 1843, Coquerel 1851, Hinton 1935, Hinton 1937, Delève 1968, Spangler and Santiago-Fragoso 1992, Spangler and Staines 2004, Maier 2013, Maier and Short 2014, Passos et al. 2010, Shepard and Chaboo 2015,) and our data, we illustrated the current distribution of all *Hexanchorus* species (Fig. 36). Specimens of the genus can be found also in El Salvador (Gutiérrez-Fonseca 2010), possibly in Chile (Vera Solís 2012) and certainly in several other countries, but we used only published species distribution data. Records of two species (*H.caraibus*, *H.gracilipes*) from Rio de Janeiro State, Brazil (Passos et al. 2010) are clearly too distant from their known distribution area including the type locality, and due to potentially erroneous determination these data were not considered. The distributional data showed that *Hexanchorus* is widely distributed from as far north as Mexico to southern Brazil. Most species are concentrated in Central America, while southern regions, including Peru, Bolivia, Paraguay or Argentina are covered by a single species records only. This indicates that for a comprehensive survey of the *Hexanchorus* diversity and distribution, intensive exploration of mainly southern areas would be required.

The revision of *Hexanchorus* material from a few localities in Ecuador and summary of published information clearly show that we still know very little about this genus. The differences in molecular distances among species and its incongruence with morphological differences in some cases highlight the importance of using DNA barcodes, because if combined, the morphological and molecular data improve significantly the robustness of the proposed taxonomy of Elmidae genera. Further research is greatly needed, employing conventional and modern techniques to better understand the true diversity of the Neotropic riffle beetles.

## Supplementary Material

XML Treatment for Hexanchorus cordillierae

XML Treatment for Hexanchorus virilis

XML Treatment for Hexanchorus rostratus

XML Treatment for Hexanchorus onorei onorei

XML Treatment for Hexanchorus onorei sagittatus

XML Treatment for Hexanchorus shepardi

## Supplementary materials

Supplementary material 1 Table S1. Genetic distance among specimens and species (Kimura 2-parameter distance) using 625bp fragment of COI gene Data type: Genetic distances This dataset is made available under the Open Database License (http://opendatacommons.org/licenses/odbl/1.0/). The Open Database License (ODbL) is a license agreement intended to allow users to freely share, modify, and use this Dataset while maintaining this same freedom for others, provided that the original source and author(s) are credited.Marek Linský, Zuzana Čiamporová-Zaťovičová, Fedor Čiampor Jr
